# Is the basal area of maize internodes involved in borer resistance?

**DOI:** 10.1186/1471-2229-11-137

**Published:** 2011-10-14

**Authors:** Rogelio Santiago, Ana Butrón, Pedro Revilla, Rosa Ana Malvar

**Affiliations:** 1 Misión Biológica de Galicia, Spanish National Research Council (CSIC). Apartado 28, 36080 Pontevedra, Spain

## Abstract

**Background:**

To elucidate the role of the length of the internode basal ring (LIBR) in resistance to the Mediterranean corn borer (MCB), we carried out a divergent selection program to modify the LIBR using two maize synthetic varieties (EPS20 and EPS21), each with a different genetic background. We investigated the biochemical mechanisms underlying the relationship between the LIBR and borer resistance. Selection to lengthen or shorten the LIBR was achieved for each synthetic variety. The resulting plants were analyzed to determine their LIBR response, growth, yield, and borer resistance.

**Results:**

In the synthetic variety EPS20 (Reid germplasm), reduction of the LIBR improved resistance against the MCB. The LIBR selection was also effective in the synthetic variety EPS21 (non-Reid germplasm), although there was no relationship detected between the LIBR and MCB resistance. The LIBR did not show correlations with agronomic traits such as plant height and yield. Compared with upper sections, the internode basal ring area contained lower concentrations of cell wall components such as acid detergent fiber (ADF), acid detergent lignin (ADL), and diferulates. In addition, some residual 2,4-dihydroxy-7-methoxy-(2H)-1,4-benzoxazin-3-(4H)-one (DIMBOA), a natural antibiotic compound, was detected in the basal area at 30 days after silking.

**Conclusion:**

We analyzed maize selections to determine whether the basal area of maize internodes is involved in borer resistance. The structural reinforcement of the cell walls was the most significant trait in the relationship between the LIBR and borer resistance. Lower contents of ADF and ADL in the rind of the basal section facilitated the entry of larvae in this area in both synthetic varieties, while lower concentrations of diferulates in the pith basal section of EPS20 facilitated larval feeding inside the stem. The higher concentrations of DIMBOA may have contributed to the lack of correlation between the LIBR and borer resistance in EPS21. This novel trait could be useful in maize breeding programs to improve borer resistance.

## Background

**Figure 1:**
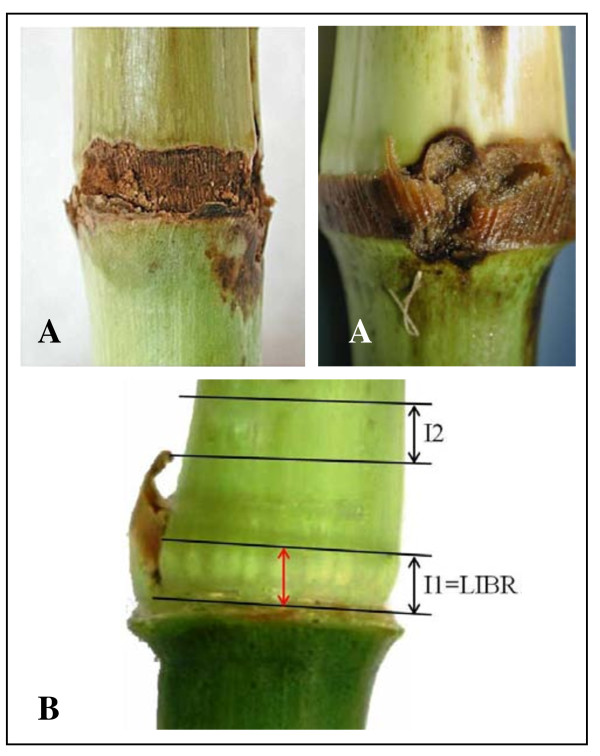
**LIBR measurement and borer damage in the area**. A- Examples of damage caused by larval feeding in internode basal area. B - Length of the basal internode ring (LIBR). LIBR measurement on one side of the internode (arrow in red), and sampling area for biochemical analyses: I1, basal part of internode = LIBR; I2, upper part of the internode (2 cm up from the LIBR area).

In the Mediterranean area, the Mediterranean corn borer (MCB), *Sesamia nonagrioides *(Lefèbvre) (Lepidoptera: Noctuidae) is a major insect pest of maize [1,2]. For this insect, the number of generations per year depends on the region, as it is affected by climate and latitude. In northwestern Spain, MCB usually has two generations per year [3]. After completing the first generation, moths of the second generation deposit their egg mass onto corn plants between the leaf sheath and the stem, usually on the internodes below the main ear [4]. After hatching, the young larvae move toward the lower part of the internode while they feed on the sheath. At node height, larvae enter the plant and feed inside the stem, producing tunnels. The nodes and their surrounding area are the preferred entry points for MCB larvae [5] (Figure 1a).

There is a large body of evidence that the morphological characteristics and structural defenses of plants affect normal feeding and establishment of corn borers on maize plants [6,7]. Several plant characteristics are associated with resistance, including general plant traits such as plant age and plant and ear height [8-11]; leaf traits such as leaf age, timing of vegetative phase transition [12,13], presence or density of trichomes [14], and leaf toughness [15-17]; stem traits such as the rind and pith toughness and thickness [5,18]; and ear traits such as husk tightness and dimension of the silk-channels [19,20]. There have been several studies on the structural characteristics of stems as mechanisms of resistance to MBC [5,18]. The rind-puncture resistance evaluated by Butrón et al. [5] was a useful indicator of resistance in some materials, but the length of the meristematic area, an area located at the base of the internode, was a more promising trait [18].

To describe that trait in more detail, the internodes in corn are formed by intercalary meristems located at the base of the internode on the upper side of a node. Within a growing internode, the younger, undifferentiated tissues are near the intercalary meristem at the base of the internode and become progressively more developed and mature higher up the internode [21]. During development, internodal cells undergo rapid elongation and the pulvinus line develops from the remains of the intercalary meristem at the base of the internode [22]. The trait recorded in previous studies as 'length of meristematic area' corresponds to the area at the base of the internode where the rind tissue is light green or white in contrast to the darker green color of the rest of the internode [23]. For accuracy, we have renamed this trait in the present study, because the relationship between the external measurement made at the end of vegetative development and the internal localization of intercalary meristem is unknown. The correct term for this measurement is 'length of the internode basal ring' (LIBR), and refers to the area located between the node and the pulvinus line (Figure 1b).

Taking into consideration this change in nomenclature, a remarkable difference in LIBR was found between inbred lines that were susceptible and resistant to MCB [18]. The susceptible inbreds showed the largest LIBR, suggesting that the size and properties of this area could be related to the ability of the larvae to enter the plant. Furthermore, this character was strongly related to stem damage, measured as tunnel length. However, as the authors pointed out, the diverse genotypes evaluated could also have other resistance mechanisms, and the correlation between the LIBR and borer resistance could be due to some other reason. Therefore, it is necessary to further examine this relationship in the same genetic background.

In the Misión Biológica de Galicia [Spanish National Research Council (CSIC)] we have developed two maize synthetic varieties with different genetic backgrounds. EPS20 belongs to the "Reid" germplasm, which is used extensively for maize breeding in temperate areas. EPS21 has a diverse "non-Reid" background. In agronomic and molecular contexts, EPS20 is more uniform than EPS21 [24,25]. After checking the variability of the LIBR in these two synthetic varieties, we carried out a divergent selection program to lengthen or shorten this area in both synthetics. Three cycles of divergent selection were carried out for each synthetic variety.

We investigated the biochemical mechanisms underlying the relationship between the LIBR and borer resistance. One of the major factors in the resistance of maize to several insects is a hydroxamic acid, 2,4-dihydroxy-7-methoxy-(2H)-1,4-benzoxazin-3-(4H)-one (DIMBOA) [26]. When present at high levels during early stages of maize development, this acid inhibits feeding by the European corn borer (ECB) O*strinia nubilalis *(Hübner) (Lepidoptera: Pyralidae) [27], and is also effective against the MCB [28,29]. However, DIMBOA concentrations decrease as the plant grows, and so this mechanism cannot protect plants against the second generation of both insect species [30]. Because the LIBR includes an area with the remains of meristematic activity and cells in a primary physiological state, it is possibly that some DIMBOA remains in this area at advanced developmental stages.

We quantified diverse cell wall compounds previously related with borer resistance in the LIBR and surrounding areas. Cell wall composition may affect insect feeding for both nutritional and physical reasons [31]. In grasses, hydroxycinnamic acids, namely *p*-coumaric (PCA) and ferulic acid (FA), are ester and/or ether-linked to cell-wall polymers [32,33]. Formation of diferulates (DFAs) and higher oligomers of FA can cross-link arabinoxylan chains [34]. These cell wall hydroxycinnamic acids in assorted tissues (kernel, leaf, pith, rind, and nodes) are related to resistance to borers including the ECB [16], the MCB [35,36], Southwestern corn borer (*Diatraea grandiosella *Dyar) (Lepidoptera: Pyralidae), and sugarcane borer (*Diatraea saccharalis *Fabricius) (Lepidoptera: Pyralidae) [37]. In addition, acid detergent fiber and lignin in maize leaf-sheaths and stalks are associated with resistance to stalk-tunneling by the ECB [38,39].

In summary, the specific objectives of the current study were as follows: (1) to determine whether the LIBR could be modified via a selection program in two genetic backgrounds; (2) to evaluate the efficacy of a selection program in terms of resistance to MCB and other agronomic traits; and (3) to elucidate the biochemical mechanisms underlying the relationship between the LIBR and borer resistance.

## Methods

### Synthetic Varieties

Eight inbred lines originating from the US Corn Belt population "Reid" and eight inbreds that were unrelated to the "Reid" population were the base materials for the synthetic varieties EPS20 and EPS21, respectively (Table 1). The synthetic variety EPS20 was formed from inbred lines derived from B14 or WF9, both of which originated from the population "Reid" [40,41]. B14 originated from the Iowa Stiff Stalk Synthetic (BSSS) that combines 16 inbred lines with resistance to stalk breakage, while WF9 was derived from the open-pollinated variety Reid Yellow dent (an Indiana Station strain). The synthetic variety EPS21 has a more heterogeneous background formed by Spanish, Italian, and French flints, and two "non-Reid" Corn Belt inbred lines.

**Table 1:** Base materials (inbred lines) for the synthetic varieties EPS20 and EPS21, and their pedigrees

Synthetic	Inbred line	**Pedigree**^**a**^	**Group of germplasm**^**b**^
**EPS20**	CM109	(V3 × B14) B14	Reid-B14
	CM139	(V3 ×B14) B14	Reid-B14
	CM151	(Mt42 × WF9) WF9	Reid-WF9
	A634	(Mt42 × B14) B14^3^	Reid-B14
	A639	A158 × B14	Reid-B14
	A652	A90 × WF9	Reid-WF9
	A664	(ND203 × A636) A636^2^	Reid-B14
	W64A	WF9 × C.I. 187-2	Reid-WF9
			
**EPS21**	EP17	A1267	Spanish flint
	EP43	Parderrubias^c^	Spanish flint
	EP53	Laro^c^	Spanish flint
	PB60	Nostrano dell'Isola^c^	Italian flint
	PB130	Rojo Vinoso de Aragón^c^	Spanish flint
	F473	Doré de Gomer^c^	French flint
	CO125	Wisc. Exp. Single cross	Corn Belt (USA)
	A509	A78 × A109	Corn Belt (USA)

^a ^Pedigrees for the US inbreds are as reported by Gerdes et al. (1993).

^b ^B14 and WF9 are two inbred lines originating from the "Reid" population, and are origins of two groups of germplasms within the Reid materials.

^c ^Local European maize varieties.

### Divergent Selection Procedure for LIBR in Both Synthetic Varieties

Divergent masal selection on both sexes was carried out in each synthetic variety (additional file 1). In 2003, approximately 600 plants were shoot-bagged for selection. When approximately 90% of the plants had been shoot-bagged, the length of the internode basal ring (LIBR) was measured in the third internode above ground level. The LIBR refers to the area located between the node and the pulvinus line in the internode, as shown in Figure 1b. Following the methodology of Santiago et al. [18], the sheath over the tissue was partially removed to measure this area. The LIBR (in mm) was measured in all normal plants, and each individual plant was labeled. According to cut-off points in either direction of selection, plants with lower values for LIBR were randomly mated to obtain the first cycle for short-length of the internode basal ring (Short_LIBR) and plants with higher values for LIBR were randomly mated to obtain the first cycle for large-length of the internode basal ring (Large_LIBR). Selection was set to apply 10% selection intensity. In 2004, second selection cycles were obtained from Short_LIBRC1 and Large_LIBRC1. In the Short_LIBRC1, plants with short LIBR were selected and mated to obtain Short_LIBRC2; in the Large_LIBRC1, plants with large LIBR were selected and mated to obtain Large_LIBRC2. In 2005, third selection cycles from Short_LIBRC2 and Large_LIBRC2 were conducted in the same way as that described above.

### Evaluation of the LIBR Response in the Selection Program

Seeds for this study were renewed by intermating at least 100 plants from each of the six cycles of selection (Large_LIBRC1, Large_LIBRC2, Large_LIBRC3 and Short_LIBRC1, Short_LIBRC2, Short_LIBRC3) and the original cycles of EPS20 and EPS21 in 2006. Field experiments for evaluations were conducted at Pontevedra (42°24' N, 8°38' W, 20 m above sea level) in 2007 and 2008. For field experiments, plants were grown in a randomized complete block design with three replications. Each plot had two rows spaced 0.80 m apart and each row consisted of 25 two-kernel hills spaced 0.21 m apart. After thinning to one plant per hill, plant density was approximately 60,000 plants ha ^-1^. The soil type is acid sandy loam. Trials were irrigated as necessary, and cultivation operations, fertilization, and weed control were carried out according to local practices. To accurately define the silking time of each genotype, plots were checked until 50% of plants showed silks. At silking, 10 plants were infested with a mass of approximately 40 MCB eggs, reared as described by Eizaguirre and Albajes [42]. At 30 days after silking, another 10 plants where evaluated to determine the characteristics of the LIBR, and plant and ear heights. The LIBR was measured as described above. Ear and plant heights were measured as the distance from the soil to the ear attachment node and to the collar of the flag leaf, respectively. At harvest, ears of infested plants were collected and kernel damage was scored on a nine-point subjective scale, as follows: 1 = 91 to 100% damage; 2 = 71 to 90%; 3 = 61 to 70%; 4 = 51 to 60%; 5 = 41 to 50%; 6 = 25 to 40%; 7 = 11 to 24%; 8 = 1 to 10%, and 9 = without damage. The stems of these plants were split into two longitudinal parts and the length of tunnels produced by larval feeding was measured (cm). The yield of uninfested plants (second row of each plot) was recorded, adjusted to kernel moisture of 140 g H_2_O kg^-1 ^and expressed as Mg ha^-1^.

### Biochemical Analysis of LIBR and Upper Sections

Original C0, Large_LIBRC3, and Short_LIBRC3 of each synthetic variety were grown at Pontevedra (42°24' N, 8°38' W, 20 m above sea level) in 2009. The field experimental design was a randomized complete block design with three replicates. Each plot had two rows spaced 0.8 m apart and each row consisted of 15 two-kernel hills spaced 0.21 m apart. After thinning to one plant per hill, plant density was approximately 60,000 plants ha ^-1^. Cultivation operations, fertilization, and weed control were carried out according to local practices and crop requirements.

The fourth internode above ground level was collected from 12 plants for biochemical analyses. Based on previous studies, samples for analysis were collected 30 days after silking when internode elongation had ceased [43]. For each internode, the basal parts of the internode corresponding to the LIBR area and a region 2 cm up from the LIBR area were separated into cylindrical stalk sections with a variable width (0.3-0.8 mm depending on the LIBR area). For simplicity, the LIBR area and the region above it are hereafter referred to as I1 and I2, respectively (Figure 1b). The outer rind (including the cuticle, epidermis, xylem elements, and phloem) was separated from the central pith tissue of each section. The pith tissue consisted of mostly parenchyma cells and randomly distributed vascular strands. Internode sections were frozen (-20 °C), lyophilized, and ground through a 0.75 mm screen in a Pulverisette 14 rotor mill (Fritsch GmbH, Oberstein, Germany).

#### DIMBOA Analysis

For DIMBOA analysis, ground material samples (each 100 mg) were weighed into screw-capped 15 mL polypropylene Falcon tubes and 5 mL HPLC grade methanol and 50 μL acetic acid were added. The tubes were vortexed and placed in a sonicator waterbath for 60 minutes at 60°C. The supernatant (0.5 mL) was combined with 0.5 mL distilled water in a microcentrifuge tube, vortexed, and centrifuged for 5 min at 1000 g. The supernatants were transferred into vials for analysis by HPLC. Analyses were performed using a 2690 Waters Separations Module (Waters, Milford, MA, USA) equipped with a 996 Photodiode Array Detector (Waters) with a Waters YMC ODS-AM (Waters, Milford, MA, USA) narrow bore column (100 × 2 mm i.d.; 3 μM particle size). For elution, the mobile phase system consisted of acetotrinile (Solvent A) and trifluoroacetic acid (0.05%) in water (solvent B) delivered in the following gradient conditions: initial A: B ratio of 10:90, changing to 30:70 in 3.5 min, then to 32:68 in 6.5 min, then to 100:0 in 4 min, then isocratic elution with 100:0 for 4.5 min, finally returning to the initial conditions after 3 min. The mobile phase flow rate was 0.3 mL/min and the total analysis time was 21.5 min. The sample injection volume was 4 μL, and the elution profiles were monitored on-line by UV absorbance at 325 and 254 nm. Retention times were compared with those of freshly prepared standard solutions. The DIMBOA standard was kindly provided by Dr. Carlos Souto from Vigo University.

#### Analysis of Hydroxycinnamic Acids

Ground material (500 mg) was extracted in 30 mL 80% methanol and mixed with a Polytron mixer (Brinkman Instruments, Westbury, NY). Samples were extracted for 1 h and then centrifuged for 10 min at 1000 g. The remaining pellet was then shaken in 20 mL 2 N NaOH under nitrogen flow for 4 h. Digested samples were neutralized with 6 N HCl, and the pH was adjusted to 2.0. After centrifugation, the supernatant was collected and the pellet washed twice with distilled water (10 mL each). Supernatants were pooled and then extracted twice with ethyl acetate (40 mL each). Collected organic fractions were combined and reduced to dryness using a Speed Vac (Savant Instruments, Holbrook, NY). The final extract was dissolved in 1.5 mL methanol and stored at -20°C prior to HPLC analysis according to the method described by Santiago et al. [35].

Retention times and UV spectra were compared with those of freshly prepared standard solutions of PCA and FA (Sigma, St. Louis, MO), and 5-5-DFA, the latter kindly provided by the laboratory of Dr. J.T. Arnason (University of Ottawa, Ontario, Canada). The UV spectra of other DFAs were compared with previously published spectra [44]. We identified and quantified four isomers of DFA: 5-5' DFA, 8-5' DFA (sum of 8-5'-non cyclic and 8-5'-benzofuran forms), and 8-o-4' DFA. The role of DFAs in resistance was based on the DFA total content (DFAT), which is commonly related to cell wall strength [34].

#### Acid Detergent Fiber (ADF) and Acid Detergent Lignin (ADL) Analyses

Fiber is composed largely of cellulose, hemicellulose, and lignin, which are the primary components of plant cell walls. ADF is composed of mostly cellulose and lignin, while ADL is primarily lignin [45]. Determinations of ADF and ADL were carried out using the AOAC Official Method 973.18: "Fibre (Acid detergent) and lignin (H_2_SO_4_) in animal feed" [46].

### Statistical Analyses

Combined analyses of variance (over years and synthetic varieties) (ANOVA) for LIBR, MCB damage, and other agronomic traits were conducted using the PROC GLM routine of SAS [47]. The sources of variation were years, replications within years, cycles of selection of synthetic varieties, and their interactions. All sources of variation, except for synthetics and cycles of selection, were considered random. The genetic progress of selection in each synthetic line was estimated by the linear regression coefficients of the LIBR plotted against cycles of selection. Progress up and down from the original cycles was estimated using the model proposed by Eberhart [48]. For each synthetic variety, sums of squares of cycles were partitioned into sums of squares due to linear and quadratic regressions and deviations from the model. Furthermore, sums of squares for linear and quadratic regressions were partitioned into average regression, and between regressions. This analysis is appropriate when two or more populations are developed from the same base population by different methods of selection, as in our study, where we compared short and large LIBRs. Estimates of average linear and quadratic coefficients for both selection directions were also calculated using the Eberhart model [48]. Simple linear regression coefficients of LIBR plotted against tunnel length and several other traits of economic importance were determined using the PROC REG routine of SAS [47].

For biochemical analyses, we combined cycles of selection of synthetic varieties and sections to compare data on the contents of diverse compounds by least significant differences (LSD) tests. All analyses were performed using the SAS program [47].

## Results and Discussion

### Responses to LIBR Selection and Relationship with Borer Resistance

**Figure 2:**
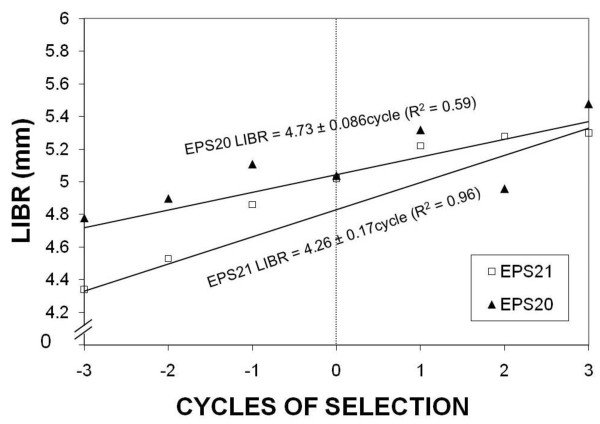
**Genetic progress of selection**. Genetic progress of selection to lengthen or shorten length of the basal internode (LIBR) in the synthetic varieties EPS20 and EPS21 estimated by linear regression coefficients.

The progress of selection for quantitative traits is usually assumed to be linear during early cycles of selection. If we consider the seven cycles of selection, the linear regression coefficients were 0.086 (P = 0.044, R^2 ^= 0.59) and 0.17 (P = 0.0005, R^2 ^= 0.93) in the synthetic varieties EPS20 and EPS21, respectively (Figure 2). Consistent with these results, a previous study on the genetic properties of the LIBR in a set of four maize inbred lines showed that additive effects were very important, and predicted that a selection program could be successful to improve the properties of the LIBR [49].

As estimated by the Eberhart model, the progress of selection for larger LIBR (b_11_) in the maize synthetic EPS20 was 0.074 mm per cycle (P = 0.27), whereas that for shorter LIBR (b_12_) in this synthetic was -0.10 mm (P = 0.12). Similarly, the linear progress of selection for larger LIBR (b_11_) in the maize synthetic EPS21 was 0.064 mm (P = 0.34) per cycle, whereas that for shorter LIBR (b_12_) was -0.27 mm (P = 0.001). The quadratic coefficients were non-significant. Previous studies have emphasized that the synthetic variety EPS21 displays higher genetic variability; therefore, a better linear response of this synthetic to selection was predictable [24,25]. Non-significant linear changes with three cycles of selection, except for shortening the LIBR in EPS21, suggest that multiple genes with small effects influence the phenotype of the LIBR.

In the combined analysis of variance, we detected significant differences among cycles of selection for most traits evaluated and a non-significant interaction for cycles × year (data not shown). We found significant differences between Large LIBRC3 and Short LIBRC3 in both synthetic varieties, with differences between opposite C3 cycles of 0.7 mm and 0.96 mm in EPS20 and EPS21, respectively (Table 2). Since changes in the LIBR may be associated with significant changes in other important agronomic traits, especially plant height or yield, these traits need to be measured. In this sense, there were non-significant differences between C3 large and short cycles of selection for any agronomic trait in both synthetics (Table 2). In addition, plant height and yield did not show significant coefficients of regression when plotted against the LIBR (Table 3); therefore, we do not expect significant correlations between the LIBR and height/yield responses.

**Table 2:** Means of different traits evaluated in three cycles of divergent selection to lengthen or shorten the length of the internode basal ring (LIBR) in the synthetic varieties EPS20 and EPS21

Cycles	LIBR (mm)	Tunnel length (cm)	Kernel damage (1-9)	Plant height (cm)	Ear height (cm)	Yield **(Mg ha**^**-1**^**)**
**EPS20**						
Large_LIBRC3	5.48a	21.57a	7.88b	229.9abc	92.5a	6.08a
Large_LIBRC2	4.96bcd	15.92abc	8.20ab	213.9abcdef	77.0bcd	5.36abcd
Large_LIBRC1	5.32ab	18.66abc	8.39ab	227.4abcde	89.1ab	5.74abc
C0	5.04abc	15.88abc	8.46a	236.7a	93.5a	5.89abc
Short_LIBRC1	5.11abc	17.14abc	8.27ab	219.5abcdef	76.8bcd	5.96ab
Short_LIBRC2	4.90bcd	11.68bc	8.53a	231.4ab	83.9abcd	6.11a
Short_LIBRC3	4.78cde	10.36c	8.68a	228.9abcd	89.5ab	5.74abc
**EPS21**						
Large_LIBRC3	5.30ab	14.62abc	8.54a	206.4cdef	73.3cd	5.06cd
Large_LIBRC2	5.28ab	12.72bc	8.44a	220.1abcdef	88.0abc	5.44abcd
Large_LIBRC1	5.22abc	11.84bc	8.70a	215.7abcdef	84.1abcd	5.74abc
C0	5.02abcd	12.44bc	8.60a	204.4ef	76.2bcd	5.66abcd
Short_LIBRC1	4.86bcd	13.20abc	8.28ab	205.8def	73.2cd	5.09bcd
Short_LIBRC2	4.53de	19.90ab	8.40ab	211.9bcdef	77.0bcd	5.32abcd
Short_LIBRC3	4.34e	12.28bc	8.34ab	202.4f	72.3d	4.80d
LSD	0.49	8.79	0.52	24.0	15.3	0.89

Notes: Trials were conducted in 2007 and 2008. Means within a column followed by the same lowercase letter are not significantly different.

**Table 3:** Simple linear regressions of agronomic and resistance traits on LIBR in synthetic varieties EPS20 and EPS21

Dependent variable	Intercept	b coefficient	Pr > F	**R**^**2**^
**EPS20**				
Tunnel length (cm) **	-61.15	15.15	0.001	0.91
Kernel damage (1-9) *	12.63	-0.84	0.034	0.62
Plant height (cm)	215.72	2.18	0.882	0.004
Ear height (cm)	41.58	8.75	0.504	0.09
Yield (Mg ha^-1^)	4.18	0.33	0.500	0.09
**EPS21**				
Tunnel length (cm)	26.76	-2.61	0.437	0.12
Kernel damage (1-9)	7.26	0.24	0.136	0.38
Plant height (cm)	166.94	8.63	0.254	0.25
Ear height (cm)	35.17	8.63	0.207	0.29
Yield (Mg ha^-1^)	2.92	0.48	0.208	0.29

*Significant at a probability level of P ≤ 0.05.** Significant at a probability level of P ≤ 0.01.

In terms of MCB resistance, we assumed that the LIBR region was related to resistance because this is the area of borer establishment and entry. Any progress in selection that results in changes to this area could affect resistance. The mean tunnel length in EPS20_Large_LIBRC3 (21.57 cm) was significantly greater than that in EPS20_Short_LIBRC3 (10.36 cm) (Table 2). Moreover, the simple regression coefficient of tunnel length (dependent variable) on LIBR (independent variable) was 15.15 mm in EPS20 (R^2 ^= 0.91, P = 0.001) (Table 3). These results indicated that our hypothesis was correct for EPS20, as shorter LIBR was associated with greater resistance. In addition, in the present study, the selection to shorten the LIBR in EPS20 showed a comparable improvement to that achieved via recurrent selection for resistance to MCB in EPS12 [48]. Sandoya and co-workers [50] reported a linear decrease for tunnel length of -1.80 cm cycle^-1^; similar to the tunnel length reduction between EPS20 and EPS20_Short_LIBRC3 of -1.84 cm cycle^-1^. Moreover, we were able to obtain one cycle per year in the masal selection procedure used in the present study to reduce the LIBR, whereas the recurrent selection program used by Sandoya and co-workers [50] required 3 years to complete one selection cycle.

There were non-significant differences in tunnel length between C0 (12.44 cm), Large_LIBRC3 (14.62 cm), and Short_LIBRC3 (12.28 cm) in EPS21 (Table 2). Moreover, the simple regression coefficient of tunnel length on LIBR in EPS21 was non-significant (R^2 ^= 0.12, P = 0.437) (Table 3). It may be that one or more additional resistance mechanisms in the base material of EPS21 masks the relationship between LIBR and resistance in this synthetic variety. For example, the inbred line CO125 contained high concentrations of diferulic acids--cell wall compounds related to MCB resistance [35,36,51], and the variety PB130 had thick cell walls that were positively related to MCB resistance [18]. In addition, the differences between the two synthetic varieties in terms of the biochemical composition of the LIBR could be important, as discussed below.

The Reid-line synthetic EPS20, which showed smaller responses to LIBR selection, partially because of its lower variability, was the synthetic variety that showed the greatest indirect response for tunnel length. This trend suggested a single resistance mechanism related to the LIBR in most of the inbred lines that make up EPS20. Butrón et al. [52] compared different germplasm groups, and proposed that the Reid germplasm has genetic mechanisms for stem-damage resistance to the MCB. Those mechanisms could be related to stalk-breakage resistance derived from the Iowa Stiff Stalk Synthetic [5].

Previous studies on maize found that there was no relationship between ear and stem resistance to the MCB [53,54]; therefore, the kernel damage response to MCB was not predictable at the start of these experiments. The healthiest ears were found in EPS20_Short_LIBRC3 (8.68) and EPS21_Short_LIBRC1 (8.70), while the more damaged ears were found in EPS20_Large_LIBRC3 (7.88) and EPS21_Large_LIBRC1 (8.28). Significant differences between opposite third cycles of selection were observed in the synthetic EPS20 (Table 2). In addition, the regression coefficient for kernel damage was negative (-0.84) and significant in EPS20 (R^2 ^= 0.62, P = 0.034) (Table 3). The negative value indicates that larger LIBR was associated with greater ear damage; however, according to the nine-point rating scale, the ears were barely damaged in both cycles and synthetics (8 = 1 to 10% damaged).

### Biochemical Resistance Mechanisms of the LIBR against Borer

We screened the diverse biochemical traits related to MCB attack in the basal internode area. For these analyses, we evaluated the composition of rind, which is the initial entry point for larvae, and the pith, which is the tissue upon which the larvae feed. In addition to the LIBR (I1), we also analyzed the area higher up the stem (I2) (Figure 1b).

#### Larvae Entry Point

We analyzed the biochemical composition of the rind, and found significant differences in DIMBOA, PCA, ADF and ADL contents between cycles of selection and among internodes sections (Table 4).

**Table 4:** Biochemical compounds in rind and pith of two internode sections in two synthetic varieties and their derivatives with lengthened or shortened length of the basal internode ring (LIBR)

Cycles	**Sections**^**a**^	**Biochemical compounds**^**b**^					
		
		DIMBOA	PCA	FA	DFAT	ADF	ADL
**EPS20_Rind**							
Large_LIBRC3	I1	0c	18976.7ab	4576.1a	116.1a	40.7bcde	6.8e
	I2	0c	18308.1abc	4465.3a	99.7a	43.5ab	10.0cd
C0	I1	92.1b	19524.3a	5023.3a	154.1a	37.0f	5.8e
	I2	0c	18829.2ab	4462.2a	129.8a	40.8bcde	12.5bc
Short_LIBRC3	I1	0c	17789.0bcd	4684.9a	123.8a	39.5cdef	6.4e
	I2	0c	17905.1bcd	4401.0a	99.0a	44.3a	18.8a
**EPS21_Rind**							
Large_LIBRC3	I1	217.2a	17675.6bcd	4457.9a	109.8a	38.8def	6.4e
	I2	0c	16828.2d	3988.2a	96.8a	44.4a	14.6b
C0	I1	69.9b	18505.4abc	4893.8a	129.5a	38.2ef	5.6e
	I2	0c	18869.3ab	4784.5a	111.4a	42.0abcd	10.9cd
Short_LIBRC3	I1	116.6b	17111.3cd	4584.1a	121.8a	39.5def	8.4de
	I2	0c	18095.5bcd	4744.3a	113.3a	42.8abc	10.6cd
LSD		60.8	1422.8			3.2	2.8

**EPS20_Pith**							
Large_LIBRC3	I1	0d	10493.2abcd	3710.1a	195.6d	20.7a	4.5a
	I2	0d	10358.7bcd	3390.5a	295.9abc	17.6a	3.1a
C0	I1	50.2bc	10647.9abc	3878.0a	244.0cd	19.5a	2.2a
	I2	0d	11717.4ab	3602.0a	294.5abc	19.6a	2.9a
Short_LIBRC3	I1	0d	10890.0abc	4062.8a	253.2bcd	21.0a	2.6a
	I2	0d	11962.1a	3917.7a	362.2a	19.9a	4.6a
**EPS21_Pith**							
Large_LIBRC3	I1	104.8a	9053.5de	3826.5a	272.8bc	21.6a	2.2a
	I2	0d	10657.4abc	3077.0a	284.4bc	20.6a	3.8a
C0	I1	53.4b	9749.1cde	3047.1a	317.1ab	22.9a	2.4a
	I2	0d	11376.3ab	3156.5a	232.0cd	18.5a	3.2a
Short_LIBRC3	I1	17.6cd	8556.7e	3686.1a	273.8bc	23.2a	3.3a
	I2	0d	10447.8bcd	2955.0a	257.5bcd	19.6a	1.9a
LSD		34.3	1494.9		71.9		

^a ^Section I1 = LIBR and I2 = 2 cm up from the LIBR area.

^b ^Analyses were conducted in 2009, and mean values are shown. Values for PCA, FA, DFAT and DIMBOA represent μg/g dry weight; those for ADF and ADL represent percentages (%).

We detected DIMBOA in the I1 section (LIBR area) of the original EPS21 and Short and Large C3 cycles of EPS21. However, in the EPS20 synthetic variety, we detected DIMBOA in the I1 section of only the original EPS20. The highest concentration of DIMBOA was in EPS21_Large_LIBRC3 (217 μg/g dry weight) (Table 4). It is important to note that DIMBOA was not detected in any I2 sections (2-cm up from the I1) (Table 4). Consistent with these results, previous studies on individual leaves noted high initial concentrations of hydroxamic acids, which declined rapidly as the leaf aged and expanded [55]. Attending to our previous hypothesis, the less developed cells in the LIBR section contained residual DIMBOA, while the DIMBOA concentrations decreased to zero further up the internode.

In maize, the hydroxamic acid concentration increases rapidly and reaches a maximum a few days after germination, and then decreases as the plant ages [27,55-58]. Bergvinson et al. [16] reported DIMBOA concentrations of approx. 300 μg/g dry weight in the pith, rind, and sheath tissues at early silking. In the current study, we determined DIMBOA concentrations in localized sections of the internode (I1) in mature plants at 30 days after silking, when internode elongation had ceased.

Regarding the inhibitory effect of DIMBOA on larvae, Barry et al. [57] concluded that less than 100 μg/g is insufficient for resistance to ECB and that a concentration of at least 400 μg/g would be a desirable target for ECB-resistance in a breeding program. In the current study, only the EPS21_Large_LIBRC3 contained significant quantities of DIMBOA (217 μg/g); this level may inhibit larval development (Table 4). There may be higher concentrations of DIMBOA at early silking, just after larvae hatching. The presence and amount of DIMBOA may be at least partly responsible for the lack of relationship between the LIBR and borer resistance in EPS21.

We analyzed the hydroxycinnamic acids bound in the cell walls of EPS20 and EPS21 and their cycles of selection. The major hydroxycinnamic compound was PCA, followed by FA, and DFAT (Table 4). In cells, PCA is mainly esterified to the γ -position of phenylpropanoid sidechains of S units in lignin [33,59,60]. Although very small quantities of PCA are esterified to arabinoxylans in immature tissues, most PCA accretion occurs in tandem with lignification [61,62]. FA is intracellularly esterified to the C5-hydroxyl of α-L-arabinose sidechains of xylans and deposited into primary and secondary walls [43,63,64]. During cell wall deposition and lignification, xylans are cross-linked by peroxidase-mediated coupling of ferulate monomers into a complex array of dimers and trimers, and by extensive copolymerization of these FA, into lignin [65]. Oxidative coupling of FA probably contributes to wall stiffening, lignin formation, cessation of growth, limited cell wall degradability by ruminants, and resistance to pests and diseases [16,35,36,64,66-70].

The levels of hydroxycinnamic acids detected in rind tissues were consistent with those determined in previous studies on various maize inbred lines [71]. There were greater concentrations of PCA and FA in the rind than in the pith, while DFAT concentrations showed the opposite trend [71] (Table 4). Rind tissues generally had greater concentrations of PCA and FA esters than pith tissues. This was expected, because rind vascular tissues lignify to a greater extent to support the conductive and supportive tissues of the internode [61]. PCA was the only hydroxycinnamic acid that showed significant differences between cycles of selection in the rind. In the I1 section, there were significant differences in PCA concentrations between some of the EPS20 cycles; that is, C0 contained higher levels of PCA than Short_LIBRC3 (19524.3 and 17789.0 μg/g dry weight, respectively), although the difference between the PCA contents in Large or Short cycles was insignificant (18976.7 and 17789.0 μg/g dry weight, respectively) (Table 4). There were no significant differences in PCA concentrations among the I1 sections of EPS21 cycles (Table 4). From those results, we could not conclude that PCA has a functional role in resistance of the rind internode basal ring area.

Among the literature on the evolution of cell wall hydroxycinnamic acids in maize internodes [43,61,72-74], the study by Scobbie et al. [74] is the most consistent with our results. Scobbie et al. [74] sectioned individual maize internodes into ten sections of equal length, and found that the lower three sections of the internode were significantly less developed than the remaining upper seven segments. They detected similar concentrations of esterified FA in all of the subsections of the internodes, but found progressively greater concentrations of esterified PCA in the upper internode sections of successively older internodes (progressing from top internodes down the stalk). Furthermore, Hatfield et al. [73] noted that the tissues at the top of a given internode contained more PCA than tissues in the lower part of the internode. In the current study, there were no differences in PCA and FA contents among the various rind sections. There are three points of difference between the previous studies and this study: Scobbie et al. [74] analyzed pith and rind jointly in each section, Hatfield et al. [73] mainly analyzed half-internode sections, and both evaluated single inbred lines. In the present study, we analyzed pith and rind separately for each section, the analyzed sections were from the lower half of the internode, and the genotypes used were synthetic varieties, each composed of eight inbred lines.

In the leaf sheaths, increased levels of NDF, ADF, cellulose, and lignin were reported to correspond to increased resistance to ECB feeding on that tissue [75-77]. In the current study, there were significant differences among cycles of selection for ADF and ADL (Table 4). In the I1 section of EPS20, there were significant differences in ADF; C0 contained 37.0% ADF and Large _LIBRC3 contained 40.7%. However, the difference in ADF between Large and Short cycles was not significant (40.7 and 39.5% ADF, respectively). No significant differences in ADF in the I1 section were found in EPS21 synthetic variety (Table 4). Furthermore, there were no significant differences in ADL in the I1 section in any of the maize synthetics or selection cycles (Table 4).

However, there were differences in ADF and ADL between the two rind sections (Table 4). The I2 sections contained higher concentrations of ADF and ADL than I1 sections. This result was consistent with previous studies showing progressively greater lignin concentrations from the base to the top of internode sections [73,74]. This reflects the greater lignin content and higher degree of lignification in the more mature tissues/cells. In this sense, and according to our original hypothesis, the lower ADF and lignin contents in the rind of the I1 section could make this site more readily penetrable by the larvae. There were no differences in ADF or ADL between Short and Large cycles of selection. However, in Large_LIBRC3, the larger area with lower ADF and ADL content could increase its susceptibility to borer entry. Conversely, the shorter LIBR in EPS20_Short_LIBRC3 could result in higher resistance by decreasing the size of the larval entry area. Nevertheless, the role of this mechanism in other genetic backgrounds, such as in the synthetic EPS21, could be obscured by other traits, such as the presence of DIMBOA as described above, or other factors.

#### Tissues Consumed by Larvae

In the pith, we observed differences in DIMBOA, PCA, and DFAT concentrations between cycles of selection (Table 4). The DIMBOA concentrations in the pith were lower than those in rind tissues, and the differences were similar to those observed in the rind. That is, we detected DIMBOA in the I1 section of the original EPS21 and EPS20, and in the Short and Large C3 cycles of EPS21. EPS21_Large_LIBRC3 contained a high concentration of DIMBOA (217 μg/g dry weight) (Table 4). In the same way, we did not detect DIMBOA in I2 sections (Table 4). As mentioned previously, the presence and level of DIMBOA in rind and pith tissues at 30 days after silking may partly explain the lack of correlation between the LIBR and borer resistance in EPS21.

The concentrations of PCA and FA were lower in the pith than in the rind, while DFAT concentrations showed the opposite trend (Table 4). These findings are consistent with previous reports, which showed that pith tissues have a lower degree of lignification, and that DFAT has a major role as a cross-linking agent to stiffen and strengthen these tissues [34,36,71]. The lower degree of lignification is reflected by the lower levels of ADF and ADL in the pith (Table 4). In addition, no significant variations of ADF and ADL were observed among the pith sections evaluated (Table 4).

Regarding the I1 section, there were no significant differences in PCA and DFAT concentrations between cycles of selection in EPS21 and EPS20 (Table 4), although it is interesting to note that I2 sections of EPS20 contained higher concentrations of DFAT. In agreement with these results, in a study on floating rice, Azuma et al. [78,79] showed that 5-5 diferulic acid was present at the lowest level around the intercalary meristem and increased as the distance from the meristematic zone increased toward the upper part of the internode. Those results suggested that the cell wall deposition of diferulic acids is not a consequence but a cause of the cessation of cell elongation in floating rice internodes. The current study is the first to describe variations in diferulates (DFAT) between two sections of maize internodes. Diferulates were not quantified in previous studies because of a lack of reliable diferulate standards, and because of the poor recovery of these compounds using traditional analytical techniques [43,72]. The role of DFAT in borer resistance, especially that of pith tissues, is well characterized [35,36,51,71]. It is possible that DFAT has a role in cessation of growth of the maize internode in some specific backgrounds, but this should be examined more closely in future studies. On the other hand, the ubiquitous presence of DFAT in EPS21, as well as the presence of DIMBOA, suggests that these and other substances may mask the effects of the LIBR on borer resistance.

## Conclusion

In summary, the LBIR showed positive responses to selection in both of the synthetic maize varieties, EPS20 and EPS21. There was a relationship between large LIBR and decreased MCB resistance in EPS20, a more uniform germplasm derived from the US Corn Belt population "Reid". A large LIBR could increase the area in which larvae can enter the stem, while a short LIBR could decrease this area, making the plant more resistant to this pest.

Structural reinforcement of the cell walls appears to be the most significant trait involved in the relationship between the LIBR and borer resistance. Lower contents of ADF and ADL in the rind of the LIBR section facilitated the entry of larvae through this area in both synthetic varieties, while lower concentrations of DFAT in the pith LIBR sections facilitated larval feeding in EPS20. We detected the antibiotic compound DIMBOA in the LIBR section at 30 days after silking in both synthetic varieties. The higher concentrations of DIMBOA in EPS21 could be partly responsible for the lack of relationship between the LIBR and borer resistance in this variety.

These experiments using selection in two genetic backgrounds enabled us to study the relationship between the basal area of maize internodes and borer resistance. Our results suggest that synthetic varieties combining diverse germplasms could contain diverse resistance mechanisms, which can mask the role of the LIBR in borer resistance. This was demonstrated in the synthetic variety EPS21, which has the most variable background. The LIBR as a resistance trait could be useful for breeding borer-resistant genotypes in maize breeding programs, especially working with "Reid" materials.

## Authors' contributions

RS assisted with the conception and design of the study, carried out field experiments and biochemical analysis, performed data analysis, and prepared the manuscript. PR and AB assisted RS with field experiments, and revised the manuscript. RAM conceived the study, participated in its design and analysis, and revised the manuscript. All authors read and approved the final manuscript.

## Supplementary Material

Additional file 1 **Diagram of divergent selection procedure for modifying the length of the internode basal ring (LIBR)**. Diagram. Click here for file
