# Supplementary figures and images for: Is the basal area of maize internodes involved in borer resistance?
Source: BMC Plant Biol. 2011 Oct 14;11:137. doi: 10.1186/1471-2229-11-137 (PMC3206430; doi:10.1186/1471-2229-11-137)

**
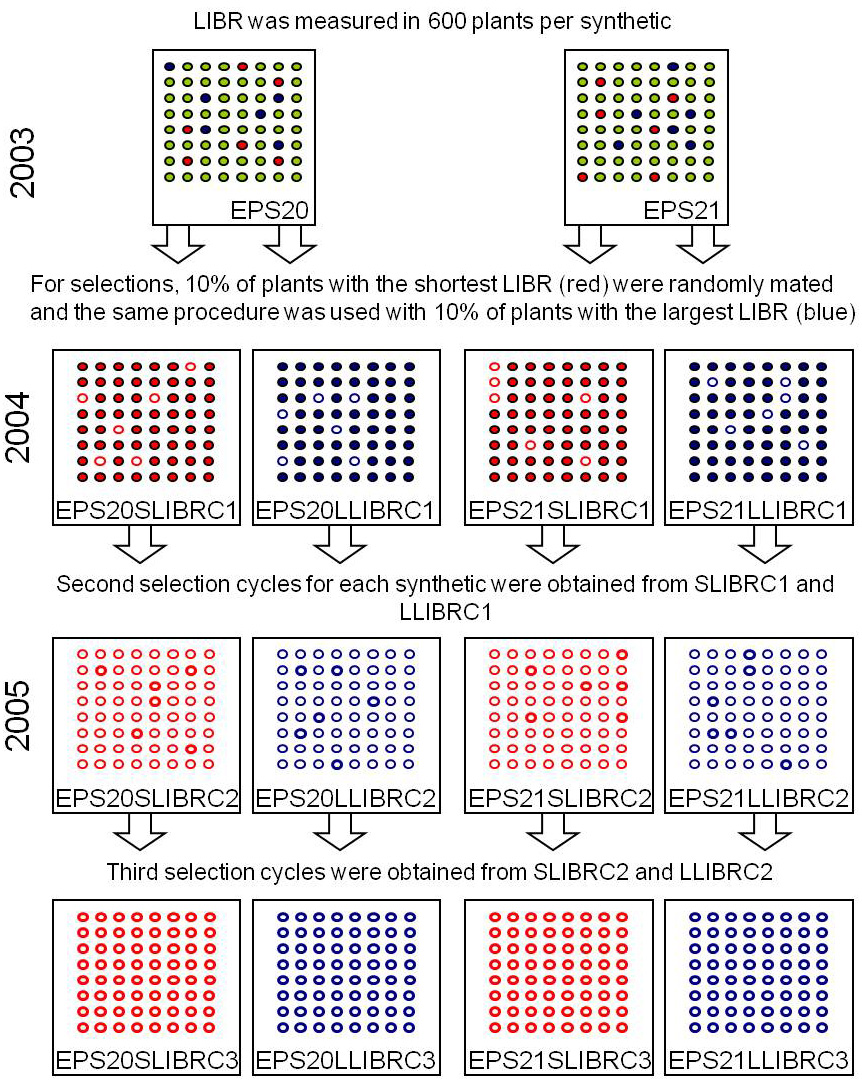
**

Supplement: Additional file 1 — Diagram of divergent selection procedure for modifying the length of the internode basal ring (LIBR). Diagram. [file 1471-2229-11-137-S1.DOC]
